# Association of Staphylococcal Populations on Teatcups of Milking Parlours with Vaccination against Staphylococcal Mastitis in Sheep and Goat Farms

**DOI:** 10.3390/pathogens10040385

**Published:** 2021-03-24

**Authors:** Charalambia K. Michael, Daphne T. Lianou, Natalia G.C. Vasileiou, Katerina Tsilipounidaki, Angeliki I. Katsafadou, Antonis P. Politis, Nikos G. Kordalis, Katerina S. Ioannidi, Dimitris A. Gougoulis, Constantina Trikalinou, Denise C. Orfanou, Ilektra A. Fragkou, Panagiota I. Kontou, Dimitra V. Liagka, Vasia S. Mavrogianni, Efthimia Petinaki, George C. Fthenakis

**Affiliations:** 1 Veterinary Faculty, University of Thessaly, 43100 Karditsa, Greece; cmichail@vet.uth.gr (C.K.M.); dlianou@vet.uth.gr (D.T.L.); agkatsaf@vet.uth.gr (A.I.K.); apolitis.vet@gmail.com (A.P.P.); nikolaoskordalis@gmail.com (N.G.K.); kate_ioan@windowslive.com (K.S.I.); dgoug@vet.uth.gr (D.A.G.); dorfanou@vet.uth.gr (D.C.O.); hfragou@vet.uth.gr (I.A.F.); dliagka@vet.uth.gr (D.V.L.); vmavrog@vet.uth.gr (V.S.M.); 2 Faculty of Animal Science, University of Thessaly, 41110 Larissa, Greece; vasileiounat@gmail.com; 3 University Hospital of Larissa, 41110 Larissa, Greece; tsilipoukat@gmail.com (K.T.); constantinatrikalinou@gmail.com (C.T.); petinaki@uth.gr (E.P.); 4 Department of Computer Science and Biomedical Informatics, University of Thessaly, 35131 Lamia, Greece; pankontou@gmail.com

**Keywords:** biofilm, goat, mastitis, milking parlour, sheep, staphylococcus, teatcup, vaccination

## Abstract

There is a paucity of information regarding staphylococcal populations on teatcups of milking parlours in sheep and goat farms. The objectives were to describe the populations of staphylococci on teatcups in milking parlours in sheep or goat farms in two field investigations throughout Greece and to potentially associate the findings with the use of anti-staphylococcal mastitis vaccinations in the farms visited during the two investigations. In a cross-sectional (255 sheep and 66 goat farms across Greece) and a longitudinal (12 sheep farms, four samplings, throughout lactation) study, swab samples were collected from 1418 teatcups (upper and lower part) for staphylococcal recovery, identification and assessment of biofilm-formation. A total of 328 contaminated teatcups (23.1%) were found in 105 sheep (41.2%) and 35 goat (53.0%) farms. Staphylococci were more frequently recovered from the upper than the lower part of teatcups: 269 *versus* 139 teatcups, respectively. After identification, 253 staphylococcal isolates were found: *Staphylococcus aureus*, *Staphylococcus equorum*, *Staphylococcus lentus*, and *Staphylococcus capitis* predominated. Of these isolates, 87.4% were biofilm-forming. The proportion of contaminated teatcups was smaller in farms where vaccination against anti-staphylococcal mastitis in general or vaccination specifically against mastitis caused specifically by biofilm-forming staphylococcal strains was applied, 19.7% or 10.9%, respectively, *versus* 25.5% in farms without vaccination. In the longitudinal study, contaminated teatcups were identified in 28 (58.3%) sampling occasions, with staphylococci being recovered more frequently from their upper part. The same species as in the cross-sectional study predominated. Of these isolates, 61.9% were biofilm-forming. In farms where vaccination against mastitis caused specifically by biofilm-forming staphylococcal strains was applied, the proportion of contaminated teatcups was smaller: 20.4% *versus* 48.3% in farms without vaccination. There were no differences in proportions of contaminated teatcups between sampling occasions. In conclusion, the great majority of staphylococci recovered from teatcups of milking parlours in sheep and goat farms included biofilm-forming isolates. Reduced staphylococcal isolation was noted in farms where anti-staphylococcal vaccination was performed; this was possibly the effect of reduced excretion of staphylococci in the milk of vaccinated animals.

## 1. Introduction

Mastitis is a significant infection of sheep and goats, adversely affecting the production of infected animals and reducing their welfare [1,2]. Staphylococci are the most frequent aetiological agents of the infection; they account for over 75% of cases of mastitis in sheep and goats. In farms, in which machine-milking takes place, the milking parlour is a location where significant transmission of staphylococci can occur [3]; this paper discussed the significance of the milking system in facilitating bacterial entry into the teat, but it did not mention relevant work on bacterial populations on the teatcups in milking parlours. Moreover, in a recent detailed literature search, it was found that the staphylococcal populations on teatcups of milking parlours have not been described [4]. In cattle farms, the presence of staphylococci on the teatcups in milking parlours has been associated with the transmission of staphylococci to cows during milking, thus leading to intramammary infections [5].

Various vaccines have been licensed and they are commercially available for prevention of mastitis in sheep or goats in the European Union. All vaccines licensed against mastitis in sheep or goats aim to prevent staphylococcal mastitis, obviously due to the importance of staphylococci as causal agents of the infection. The efficacy of these vaccines varies, but, in all cases, vaccination should not be considered to be 100% effective and it must be coupled with additional udder health management measures to improve control of the infection [6]. Whilst these vaccines are not fully effective in preventing the infection, nevertheless, after their administration, a marked decrease in the shedding of staphylococci from vaccinated animals has been described, even after experimental intramammary infections [6,7].

The first objective of this study was to describe the populations of staphylococci on teatcups in milking parlours in two investigations in sheep or goat farms throughout Greece. The second objective of the study was to potentially associate the findings of staphylococci on teatcups with the application of anti-staphylococcal mastitis vaccinations in the farms that were visited during the investigations.

## 2. Results

### 2.1. Cross-Sectional Study

#### 2.1.1. Isolation of Staphylococci from Teatcups in Milking Parlours

Contaminated teatcups were found in the milking parlours of 105 sheep flocks (41.2%; 95% CI: 35.3–47.3%) and 35 goat herds (53.0%; 95% CI: 41.2–64.6%) (*p* = 0.08).

There were 241 contaminated teatcups in sheep flocks (21.6%; 95% CI: 19.3–24.1%) and 87 contaminated teatcups in goat herds (28.7%; 95% CI: 23.9–34.1%) (*p* = 0.009); i.e., in total, 328 contaminated teatcups (23.1%; 95% CI: 21.0–25.4%) were found. Staphylococci were recovered significantly more frequently from the upper than the lower part: in 269 *versus* 139 teatcups, respectively (sheep: 199 *versus* 97, goats: 70 *versus* 42; *p* < 0.001 for all of the comparisons) (Table 1).

Cumulatively, 425 staphylococcal isolates were recovered from the samples (i.e., 1.3 staphylococcal isolates per teatcup): 309 from teatcups in sheep parlours and 116 from teatcups in goat herds (i.e., on average, 1.28 and 1.33 per contaminated teatcup, respectively; *p* = 0.28). Of these, 279 (65.6%) were recovered from the upper and 146 (34.4%) from the lower part of the teatcups sampled; no significant difference was seen between the average isolations per contaminated sample: 1.03 *versus* 1.05, respectively; *p* = 0.41).

#### 2.1.2. Identity of Staphylococci Isolated from Teatcups

253 staphylococcal isolates were found after identification: 186 in sheep flocks (0.73 isolate per parlour) and 67 in goat herds (1.02 isolates per parlour) (*p* = 0.06). Significantly more staphylococcal isolates were found in the samples from the upper than the lower part of teatcups: 192 *versus* 114 isolates (i.e., 0.60 *versus* 0.36 isolate per parlour) (*p* < 0.001).

The isolates were classified into a total of 25 staphylococcal species. Of the isolates, 21 (8.3%) were *Staphylococcus aureus*, one (0.4%) was *S. intermedius,* and the other 231 (91.3%) were coagulase-negative species. Among these, the most frequently identified species was *S. equorum* (*n* = 44, 17.4%) (Table 2). *S. aureus* was recovered more frequently from the lower than the upper part of contaminated teatcups: 15 (13.2%) *versus* 12 (6.3%) isolates (*p* < 0.001). In contrast, coagulase-negative isolates were more frequently recovered from the upper than the lower part of the contaminated teatcups: 180 (93.7%) *versus* 98 (86.8%) (*p* < 0.001). Among coagulase-negative species, no differences were seen in the frequency of isolation from the upper or lower part of contaminated teatcups for the five species more frequently recovered, namely for *S. equorum*: 37 (20.7%) *versus* 13 (13.1%) isolates (*p* = 0.12), for *S. lentus*: 27 (15.1%) *versus* 18 (18.2%) isolates (*p* = 0.50), for *S. haemolyticus*: 17 (9.5%) *versus* 11 (11.1%) (*p* = 0.69), for *S. capitis:* 16 (10.6%) *versus* 10 (10.1%) (*p* = 0.75), and for *S. sciuri*: 13 (7.3%) *versus* 10 (10.1%) (*p* = 0.41).

#### 2.1.3. Biofilm-Formation by Staphylococci Isolated from Teatcups

In total, the biofilm-forming staphylococci were recovered from 290 teatcups (88.4% of contaminated teatcups; 95% CI: 84.5–91.4%). There was no difference between sheep flocks and goat herds or upper and lower part of teatcups in the proportion of recovery of biofilm-forming staphylococci: in all cases, this was over 85% (*p* > 0.25 for all comparisons).

Of the 253 staphylococcal isolates recovered, 221 (87.4%; 95% CI: 82.7–90.9%) were found to be biofilm-forming. The proportion of biofilm-forming isolates was not significantly different in sheep flocks and goat herds: 89.2% (166 isolates) and 82.1% (55 isolates), respectively (*p* = 0.13). However, more biofilm-forming isolates were recovered from the lower than the upper part of teatcups: 89.4% (101 isolates) and 88.5% (170 isolates), respectively (*p* < 0.001).

All of the *S. aureus* isolates (100.0%) were biofilm-forming. Among the coagulase-negative isolates, 200 (86.6%) were biofilm-forming (*p* = 0.06 for the difference in the proportions of biofilm-forming isolates among *S. aureus* and coagulase-negative staphylococci).

#### 2.1.4. Association of the Recovery of Staphylococci from Teatcups with the Application of Anti-Mastitis Vaccination

Vaccination against staphylococcal mastitis was performed in 107 sheep flocks (42.0%; 95% CI: 36.1–48.1%) and 28 goat herds (42.4%; 95% CI: 31.2–54.4%) (*p* = 0.53).

There was a tendency for contaminated teatcups to be found in fewer farms with vaccination than in farms without it: 37.8% (51/135; 95% CI: 30.1–46.2%) *versus* 47.9% (89/186; 95% CI: 40.8–55.0%) of respective farms (*p* = 0.07). However, in farms with vaccination, the proportion of contaminated teatcups was significantly smaller than in farms without it: 19.7% of teatcups in farms with vaccination (116/588; 95% CI: 16.7–23.1%) *versus* 25.5% of teatcups in farms without vaccination (212/830; 95% CI: 22.7–28.6%) (*p* = 0.011). This significance was higher for the lower than the upper part of teatcups: *p* = 0.013 *versus p* = 0.037, respectively. The average proportion of contaminated teatcups within farms was 20.8% in farms with vaccination *versus* 25.6% in farms without vaccination (Table 3).

In the univariable analysis, there was no association between the management system that was applied in the farms and the proportion of contaminated teatcups therein: 18.2% for farms with intensive management, 23.0% for farms with semi-intensive management, and 23.7% for farms with semi-extensive or extensive management (*p* = 0.18). Additionally, there was no correlation between the number of milking units in the parlour or the number of female animals in the farm and the proportion of contaminated teatcups therein (*r* = −0.0678 and 0.0199, respectively; *p* = 0.11 and 0.36).

In the multivariable analysis, only vaccination emerged with a tendency of association (*p* = 0.07) with the proportion of contaminated teatcups in the farms. Among the vaccination regimes that were recorded and the vaccines used, vaccine III was the one associated with the lower proportion of contaminated teatcups in the respective farms (Table 4). Finally, in farms with vaccination, there was a correlation between the length of the period from the start of lambing/kidding season to sampling of teatcups and the proportion of contaminated teatcups in the farm: *r* = 0.1560 (*p* = 0.035).

**Figure 1:**
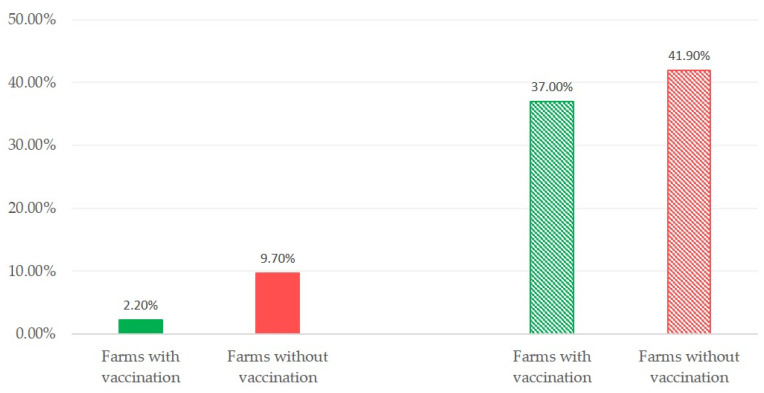
Proportions of teatcups contaminated with *S. aureus* (full pattern; *p* = 0.007 between farms) or coagulase-negative staphylococci (massif pattern; *p* = 0.38 between farms) found in vaccinated against staphylococcal mastitis (green colour) or unvaccinated (red colour) sheep or goat farms.

Of the 21 *S. aureus* isolates, three (14.3% of isolates) were recovered from farms with vaccination (2.2% of such farms) and 18 (85.7% of isolates) were recovered from farms without vaccination (9.7% of such farms) (*p* = 0.007 for the rate of recovery between farms with or without vaccination). In contrast, no such difference between farms with or without vaccination was seen for the recovery of coagulase-negative staphylococci: 37.0% versus 41.9% (*p* = 0.38) (Figure 1).

Among the vaccines used, one (vaccine III) was specifically effective against biofilm-forming staphylococci. In farms, in which vaccination with this product was performed, the proportion of teatcups contaminated with biofilm-forming staphylococci was significantly smaller: 10.9% of teatcups in farms with this specific vaccination (45/411; 95% CI: 8.3–14.3%) *versus* 25.5% in farms without this vaccination (257/1007; 95% CI: 22.9–28.3%) (*p* < 0.001). Again, this significance was higher for the lower than the upper part of teatcups: *p* = 1.90 × 10^−7^ versus *p* = 0.74 × 10^−7^, respectively (Table 5). The average proportion of contaminated teatcups within farms was 12.1% in farms with this vaccination *versus* 25.1% in farms without this vaccination. Of the staphylococcal isolates recovered from teatcups in farms with this specific vaccination, the proportion of biofilm-forming staphylococci recovered from teatcups was significantly smaller than in farms in which no such vaccination was performed: 69.2% *versus* 92.0% (*p* < 0.002).

### 2.2. Longitudinal Study

Contaminated teatcups were found, on at least one sampling occasion, in the milking parlours of 11 of the 12 sheep flocks (91.7%; 95% CI: 64.6–98.5%). Of the 48 total sampling sessions (i.e., 12 flocks × 4 samplings in each), contaminated teatcups were identified in 28 (58.3%; 95% CI: 44.3–71.2%). Staphylococci were recovered significantly more frequently from the upper than the lower part: in 44 *versus* 19 occasions, respectively (*p* < 0.001 for all of the comparisons). Cumulatively, 63 staphylococcal isolates were recovered from the samples.

After identification, 42 staphylococcal isolates were found. Significantly more staphylococcal isolates were found in the samples from the upper than the lower part of teatcups: 38 *versus* 18 isolates (i.e., 0.23 *versus* 0.11 isolate per sampling site) (*p* < 0.001).

The isolates were classified into a total of 11 staphylococcal species. Of the isolates, five (11.9%) were *S. aureus*, and the other 37 (88.1%) were coagulase-negative species. Among these, *S. equorum* (*n* = 10, 27.0%), *S. lentus* (*n* = 7, 18.9%), and *S. capitis* and *S. sciuri* (*n* = 5 each, 13.5%) were the most frequently identified species (Table S1).

Of the 42 staphylococcal isolates recovered, 26 (61.9%; 95% CI: 46.8–75.0%) were found to be biofilm-forming. Of the five *S. aureus* isolates, four (80.0%) were biofilm-forming. Among the coagulase-negative isolates, 22 (59.5%) were biofilm-forming (*p* = 0.36).

Vaccination against staphylococcal mastitis was applied in eight flocks, all with the same vaccine (vaccine III). Overall, in flocks in which vaccination was applied, the proportion of contaminated teatcups was significantly smaller: 20.4% in farms with vaccination (22/108; 95% CI: 13.9–28.9%) *versus* 48.3% in farms without vaccination (29/60; 95% CI: 36.2–60.7%) (*p* < 0.001). This significance was higher for the lower than the upper part of teatcups: *p* = 0.007 *versus p* = 0.002, respectively. Throughout the four sampling occasions, the difference in proportions of contaminated teatcups between farms with or without vaccination was also significant (*p* = 0.015); however, there were no differences in the proportions of contaminated teatcups between the four sampling occasions (*p* = 0.46) (Table 6).

## 3. Discussion

The results indicate that, despite sampling after the regular post-milking cleaning of parlours and teatcups performed in the farms, there were still staphylococci thereon. These staphylococci could have originated from the animals in the farm; additionally, they could have been of human or environmental origin. Likely, the higher recovery of staphylococci from the upper part of the teatcups was the consequence of the increased exposure of that part to external contamination by environmental isolates. In contrast, isolates that were recovered from the lower part of teatcups might have originated more often from animals, as milk during its flow out of the teat during milking comes in contact with that part of the cups.

The great majority (87%) of staphylococci included biofilm-forming isolates. Specifically for sheep farms, this proportion (89%) was higher than among staphylococci that were recovered from the bulk-tank milk of the same sheep farms (72%) [8]. Biofilm-formation by these isolates had helped them to attach on the teatcups and, thus, survive cleaning. Such bacteria can survive even during unfavourable conditions, e.g., treatment with antimicrobial chemicals. Indeed, the removal of bacterial biofilms by using standard cleaning and sanitation procedures has been found to be difficult or impossible [9].

These staphylococci might then pass to the bulk-milk at a subsequent milking. In general, bacterial biofilms can lead to various problems during food production [10,11]. For the dairy industry specifically, the presence of bacterial biofilms can raise some zoonotic concerns; for example, biofilms in milk and dairy products can become habitat for bacteria, e.g., *Listeria monocytogenes*, which are confirmed human pathogens [12,13].

Staphylococci are important causal agents of mastitis in sheep and goats [14,15]. Staphylococci, which had survived on the teatcups, can invade into the teat during the subsequent milking session and then into the mammary gland of animals, causing mastitis. The reverse pressure gradient is a mechanism by which this can occur; specifically, pressure differences that may occur for around 0.02 to 0.05 s at a level of 1.5 to 7.0 kPa in the milking system during the milking session can lead to an influx of bacteria present around the teat orifice (i.e., on the teatcups) into the teat canal [3]. As most of the isolates recovered were found to be biofilm-forming, they could easily attach on the teat skin, especially if there were skin lesions, possibly caused during machine-milking; teat lesions have been found to predispose to bacterial attachment on the teat skin and, subsequently, to bacterial invasion and mastitis [16].

Anti-mastitis vaccination was performed in 42% of the farms. Three commercially available vaccines were used. Among these vaccines, one offering protection specifically against biofilm-forming staphylococci (vaccine III) was the most frequently used (in 69% of farms in which vaccination was applied). The finding of a lower frequency of contaminated teatcups in farms in which anti-staphylococcal mastitis vaccination was applied is in line with the reduced excretion of staphylococci in the milk of vaccinated animals, as has been reported in previous studies [6,7]. The higher statistical significance of the respective results regarding the lower part of teatcups lends further support to this argument. There was also a clear significance of the results regarding *S. aureus*, an important mammary pathogen in dairy ewes and does [14,15], which was isolated less often from farms with vaccination; in contrast, no significance was seen between farms with or without vaccination with the coagulase-negative species recovered (*S. equorum*, *S. lentus*, *S. haemolyticus*, *S. capitis*, and *S. sciuri*), which are not predominant mammary pathogens, but have originated from environmental sources. Moreover, an association of the length of time after lambing/kidding was seen with the proportion of contaminated teatcups. All of these findings further support the initial hypothesis.

Nevertheless, it should be pointed out that anti-staphylococcal mastitis vaccination would only have an indirect effect of staphylococcal populations on teatcups; vaccination can limit excretion of staphylococci from vaccinated ewes [7] and the present results provide indirect evidence for that. Hence, fewer bacteria would be disseminated and, thus, would contaminate the teatcups. Exactly because this would be an indirect effect, it should be indicated that various other factors (e.g., management practices) might also influence the result in individual farms. Moreover, vaccination would have no effect in staphylococcal species that are primarily environmental organisms.

The results were stronger in farms, where the vaccine against biofilm-forming staphylococci (vaccine III) was used. This was reasonable, given that most isolates that were recovered from the teatcups were biofilm-forming. This finding may possibly indicate that such a vaccine might contribute to limiting staphylococcal dissemination to other animals at the farm during milking. This can be the result of the reduced staphylococcal excretion and isolation from animals vaccinated with that, as previously found in field and experimental studies [6,7]. The application of management practices that reduce bacterial contamination of teat ends is a prime aspect of mastitis control [17]. The reduced staphylococci in farms with vaccinated animals would further contribute to decreased risk infection of ewes and does at the milking parlour. The present results provide indirect evidence that vaccination, specifically against biofilm-forming staphylococci, contributes to a reduction of shedding of staphylococci, as found in previous relevant field studies [6,7] and the reduction of such contamination in the farm environment.

A further typing of these isolates (e.g., by means of multi-locus sequence typing [18]) can be useful in confirming their epidemiology, which, in turn, would support their effective control. Moreover, staphylococcal isolates on the teatcups are at the interface between animal and human infections. As discussed above, they may infect animals during milking. Moreover, these bacteria, during milking, can pass to the milk in the farm, which is produced for human consumption. Hence, antimicrobial resistance genes may be transferred between animals and humans by means of these organisms. Therefore, it is worth evaluating the patterns of resistance to antimicrobial agents of these isolates.

## 4. Materials and Methods

### 4.1. Farms and Sampling

**Figure 2:**
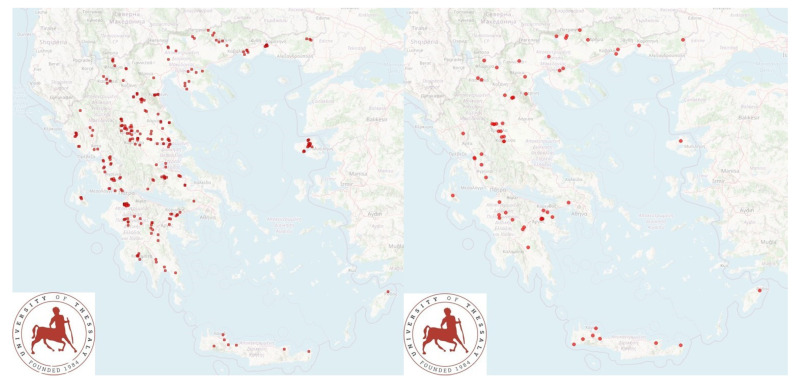
Location of 255 sheep flocks (left map) and 66 goat herds (right map) around Greece visited for swab-sampling of teatcups in the milking parlour.

In this work, a cross-sectional study was performed in 321 sheep or goat farms throughout the country (Figure 2), followed by a longitudinal study with four visits in 12 sheep farms (Tables S2 and S3).

- Cross-sectional study. In total, 255 dairy sheep flocks and 66 dairy goat herds in the 13 administrative regions of Greece were included into the study and visited for a collection of samples and information. Veterinarians that were active in small ruminant health management around Greece were contacted by telephone and asked whether they wished to collaborate in the investigation [4,8]; in total, 46 veterinarians collaborated in this work. The flocks were selected by the collaborating veterinarians on a convenience basis (willingness of farmers to accept a visit by University personnel for an interview and sample collection). The principal investigators (CKM and GCF) accompanied by other investigators (DTL, NGCV, APP, NGK, KSI, DAG, DCO, and DVL) visited the respective farms for sample collection.
- Longitudinal study. In total, 12 dairy sheep flocks selected on convenience basis were visited four times within a lactation period. The first visit was performed within 15 to 20 days after the start of the milking season. The second visit was performed two months after the first and the third two months after the second. Finally, the fourth visit was performed three months after the third, before the ewes were dried-off.

On each farm, swabbing of the teatcups of the milking parlour took place after the end of a milking session and the cleaning of the parlour, which was performed by fol-lowing the usual farm routine. This included a stage of washing of the parlour with water, to clean liners, clusters, long milk tubes, and pulse tubes, a stage of flushing with added chemicals (detergents with acid or alkaline pH) and a stage of rinsing.

Two teatcups were swabbed in parlours with one milking unit (*n* = 1 sheep and 3 goat farms), three teatcups were swabbed in parlours with two to 12 milking units (*n* = 157 sheep and 32 goat farms), six teatcups in parlours with 13 to 24 units (*n* = 81 and 28), nine teatcups in parlours with 25 to 36 units (*n* = 12 and 1), and 12 teatcups in parlours with 37 to 48 units (*n* = 4 and 2). The specific teatcups sampled in each parlour had been predetermined using an electronic random number generator. Two separate samplings were performed in each teatcup: one from the upper (approx. 1–1.5 cm deep) and one from the lower (approx. 10–12 cm deep) part of the teatcup; swabbing included the entire circumference of the inner wall of the teatcup in a circular manner. In each swabbing, duplicate samples were taken (i.e., in total, four sterile swabs were used in each teatcup). In total, 1115 teatcups were sampled on sheep farms and 303 on goat farms; therefore, a total of 5672 swab samples was cumulatively collected and processed.

The swabs were immersed into transportation medium (Liquid Based Microbiology—LBM; BioMerieux, Marcy-l’-Étoile, France). Transportation to the laboratory was made by the investigators and by car; samples were collected from farms in the islands were also transported as accompanying luggage by airplane (Crete, Lesvos and Rhodes) or by boat (Cephalonia).

### 4.2. Laboratory Examinations

Bacteriological examinations started within 24 h after the collection of samples. Each of the four swabs obtained from a teatcup was cultured in duplicate on 5% sheep blood agar and *Staphylococcus* selective medium (mannitol agar). All of the plates were incubated aerobically at 37 °C for 48 h; if there was no growth, the plates were re-incubated for another 24 h. Bacterial isolation and initial identification were performed using standard methods [19,20]. The detection of at least three confirmed staphylococcal colonies on at least one agar plate of those cultured with each swab was considered to indicate the presence of the organism. Finally, the staphylococcal isolates were identified to the species level by using Matrix-Assisted Laser Desorption/Ionization Time-of-Flight Mass Spectrometry (VITEK MS; BioMerieux, Marcy-l’-Étoile, France). In brief, isolates were smeared from Petri dishes onto target slides and then 1 μL VITEK MS matrix was applied over the sample and air-dried and allowed to co-crystallize with the sample; target slides with all of the so-prepared isolates were loaded into the VITEK MS system. Subsequently, the mass spectra of whole bacterial cell proteins were acquired and compared to the known mass spectra included in the database for each staphylococcal species.

All of the staphylococcal isolates were processed for evaluation of in vitro biofilm formation. This was tested by a combination of (a) culture appearance on Congo Red agar plates and (b) results of microplate adhesion test, as detailed by Vasileiou et al. [21] for staphylococcal isolates recovered from milk.

### 4.3. Data Management and Analysis

#### 4.3.1. Data Management

For evaluation of the staphylococci on teatcups, the two sampling sites (upper and lower part of a teatcup) were initially considered separately. In the case of staphylococcal recovery from at least one of the two sites, the teatcup was described as ‘contaminated’.

In the consideration of staphylococcal species, when the same bacterial species was identified from samples collected from the same parlour, it was considered to be the same organism and was taken into account in any relevant calculations only once.

The results of testing for biofilm formation by the staphylococcal strains that were obtained by each method were assessed. Subsequently, the results of the two methods (culture appearance on Congo Red agar and microplate adhesion) were combined [21] and staphylococcal strains were characterised as biofilm-forming or non-biofilm-forming.

During the study, it was recorded that four immunological products (vaccines) were used; three (I, II, III) were commercially available products, fully licenced in the European Union, whilst the fourth (IV) was an autogenous vaccine (Table S4). All of the vaccines had been administered to animals at the end of the preceding gestation, in order to provide protection to animals from the start of the lactation period.

The length of the period from start of the lambing/kidding season in a farm to time of the visit and swab-sampling of teatcups was estimated.

#### 4.3.2. Statistical Analysis

The data were entered into Microsoft Excel and analysed using SPSS v. 21 (IBM Analytics, Armonk, NY, USA). Basic descriptive analysis was performed. The exact binomial confidence intervals (CI) were obtained.

Frequencies were compared by using cross-tabulation with Pearson’s chi-square test, Fisher exact test, or McNemar’s test, as appropriate. One-way analysis of variance was used to analyse the differences among group means.

The outcome of ‘proportion of contaminated teatcups in farms’ was considered. The potential associations with the management system applied in farms (intensive, semi-intensive, or semi-extensive/extensive), the number of female animals in the farm (no.), the application of vaccination (no vaccination, vaccine I, vaccine II, vaccine III, or vaccine IV), and the number of milking units in the parlour (no.) were initially evaluated in univariable analysis by using cross-tabulation with Pearson’s chi-square test or analysis of correlation, as appropriate and depending on the nature of the data.

Subsequently, a multivariable model was created using mixed-effects logistic regression with farms as the random effect, and offering the above variables to the model. Variables were removed from the initial model by backwards elimination. The *p* value of removal of a variable was assessed by the likelihood ratio test, and for those with a *p* value of >0.2 the variable with the largest probability was removed. This process was repeated until no variable could be removed with a *p* value of >0.2. The final multivariable test required the following variables: (a) the application of vaccination in the farm and (b) number of milking units in the parlour.

In the cross-sectional study, an analysis of correlation was used between the length of the period from start of the lambing/kidding season in a farm to time of the visit and swab-sampling of teatcups and the proportion of contaminated teatcups in the farm.

Statistical significance was defined at *p* < 0.05.

## 5. Conclusions

Staphylococci were recovered from teatcups of sheep and goat parlours, despite the regular post-milking cleaning of parlours. The great majority of staphylococci included biofilm-forming isolates; biofilm-formation helped the bacteria to attach on the teatcups and survive the unfavourable conditions. A reduced frequency of staphylococcal recovery was noted in farms where anti-staphylococcal vaccination was performed; this was possibly the effect of reduced excretion of staphylococci in milk of vaccinated animals. This finding confirms that anti-staphylococcal mastitis vaccination can reduce staphylococcal dissemination in the farms.

## Figures and Tables

**Table 1:** The frequency of recovery of staphylococci from teatcups of milking parlours in sheep and goat farms.

	Recovery of Staphylococci from Contaminated Teatcups		
	Upper Part (*n*)	Lower Part (*n*)	Total (*n*)
Sheep flocks	199 (17.8%) ^a,d^	97 (8.7%) ^a,e^	241 (21.6%) ^f^
Goat herds	70 (23.1%) ^b,d^	42 (13.9%) ^b,e^	87 (28.7%) ^f^
Total	269 (19.0%) ^c^	139 (9.8%) ^c^	328 (23.1%)

^a–f^ pairs of proportions marked with the same superscript are significantly different between them as follows: for ^a–c^ *p* < 0.001, for ^d^ *p* = 0.039, for ^e^ *p* = 0.007, for ^f^ *p* = 0.009.

**Table 2:** Identity of staphylococcal isolates recovered from teatcups of milking parlours in sheep and goat farms and their frequency of recovery.

Sheep Flocks (*n* = 186)				Goat Herds (*n* = 67)			
Upper Part of Teatcups (*n* = 141)		Lower Part of Teatcups (*n* = 82)		Upper Part of Teatcups (*n* = 51)		Lower Part of Teatcups (*n* = 32)	
Species	*n*	Species	*n*	Species	*n*	Species	*n*
*S. arlettae*	1	*S. aureus*	10	*S. aureus*	4	*S. aureus*	5
*S. aureus*	8	*S. capitis*	5	*S. auricularis*	1	*S. capitis*	5
*S. auricularis*	2	*S. chromogenes*	4	*S. capitis*	6	*S. cohnii* s. *urealyticum*	1
*S. capitis*	10	*S. cohnii* s. *cohnii*	3	*S. caprae*	1	*S. epidermidis*	1
*S. chromogenes*	5	*S. cohnii* s. *urealyticum*	1	*S. epidermidis*	1	*S. equorum*	3
*S. cohnii* s. *cohnii*	3	*S. epidermidis*	1	*S. equorum*	8	*S. haemolyticus*	6
*S. epidermidis*	2	*S. equorum*	10	*S. haemolyticus*	4	*S. kloosii*	1
*S. equorum*	29	*S. gallinarum*	2	*S. kloosii*	1	*S. lentus*	3
*S. haemolyticus*	13	*S. haemolyticus*	5	*S. lentus*	5	*S. pasteuri*	1
*S. hominis*	2	*S. hominis*	2	*S. pasteuri*	3	*S. pettenkoferi*	1
*S. intermedius*	1	*S. kloosii*	1	*S. pettenkoferi*	2	*S. saprophyticus*	3
*S. kloosii*	2	*S. lentus*	15	*S. saprophyticus*	6	*S. sciuri*	1
*S. lentus*	22	*S. pettenkoferu*	1	*S. sciuri*	2	*S. warneri*	1
*S. pasteuri*	6	*S. saprophyticus*	3	*S. simulans*	2		
*S. pettenkoferi*	2	*S. sciuri*	9	*S. warneri*	4		
*S. saprophyticus*	7	*S. vitulinus*	2	*S. xylosus*	1		
*S. schleiferi* s. *schleiferi*	1	*S. warneri*	5				
*S. sciuri*	11	*S. xylosus*	3				
*S. simulans*	1						
*S. vitulinus*	1						
*S. warneri*	7						
*S. xylosus*	5						

s.: subspecies.

**Table 3:** Proportions of contaminated teatcups (with staphylococci) in milking parlours in sheep and goat farms, according to anti-staphylococcal mastitis vaccine applied.

Vaccination	Farms (*n*)	Recovery From	
		Upper Part of Teatcups	Lower Part of Teatcups
Vaccine I	16	14.3% (9/63) ^a^	15.9% (10/63) ^b^
Vaccine II	25	32.4% (35/108) ^a^	13.0% (14/108) ^b^
No vaccination	186	20.7% (172/830) ^a^	11.4% (95/830) ^b^

^a,b^ groups of proportions marked with the same superscript are significantly different between them as follows: for ^a,b^ *p* < 0.001.

**Table 4:** Results of multivariable analysis for effect of vaccination and vaccine used against staphylococcal mastitis in the proportion of contaminated teatcups (with staphylococci) in the milking parlours of 321 sheep and goat farms in Greece (mixed effects logistic regression).

Vaccination	Proportion of Contaminated Teatcups	Odds Ratio (95% Confidence Intervals)	*p*
No vaccination	25.5% (212/830)	2.088 (1.518–2.871)	<0.001
Vaccination with vaccine I	23.8% (15/63)	1.902 (1.000–3.617)	0.050
Vaccination with vaccine II	37.0% (40/108)	3.580 (2.217–5.781)	<0.001
Vaccination with vaccine III	14.1% (58/353)	reference	
Vaccination with vaccine IV	50.0% (3/6)	6.086 (1.199–30.885)	0.029

**Table 5:** The proportions of contaminated teatcups (with biofilm-forming staphylococci) in milking parlours in sheep and goat farms, according to application of vaccination with a vaccine specific against mastitis against biofilm-forming staphylococci.

Vaccination	Farms (*n*)	Recovery From	
		Upper Part of Teatcups	Lower Part of Teatcups
Vaccine III	93	9.7% (40/411) ^a^	2.7% (11/411) ^b^
No vaccination against biofilm-forming staphylococci	228	20.8% (209/1007) ^a^	11.3% (114/1007) ^b^

^a,b^ pairs of proportions marked with the same superscript are significantly different between them as follows: for ^a,b^ *p* < 0.001.

**Table 6:** The proportions of contaminated teatcups (with staphylococci) in milking parlours in sheep flocks throughout a lactation period, according to anti-staphylococcal mastitis vaccine applied.

Vaccination	Farms (*n*)	Sampling Occasion			
		1st	2nd	3rd	4th
Vaccine III	8	4.6% (5/108) ^a^	4.6% (5/108) ^a^	5.6% (6/108) ^a^	5.6% (6/108) ^a^
No vaccination against biofilm-forming staphylococci	4	8.3% (5/60) ^a^	15.0% (9/60) ^a^	13.3% (8/60) ^a^	11.7% (7/60)^a^

^a^ frequencies marked with the same superscript are significantly different between them as follows: for ^a^ *p* < 0.02.

## Data Availability

Most data presented in this study are in the manuscript. The remaining data are available on request from the corresponding author. The data are not publicly available as they form part of the PhD thesis of the first author, which has not yet been examined, approved and uploaded in the official depository of PhD theses from Greek Universities.

## Supplementary Materials

The following are available online at https://www.mdpi.com/2076-0817/10/4/385/s1, Table S1: Identity of staphylococcal isolates recovered from teatcups of milking parlours in sheep flocks (*n* = 12) during a longitudinal study (4 visits) and their frequency of recovery, Table S2: Summary of characteristics of 321 farms included into a cross-sectional study in Greece regarding staphylococcal populations on teatcups of milking parlours, Table S3: Summary of characteristics of 12 sheep farms included into a longitudinal study in Greece regarding staphylococcal populations on teatcups of milking parlours, Table S4: Details of vaccines recorded to be in use in Greece against staphylococcal mastitis, during a countrywide investigation in sheep and goat farms.
