# Supplementary material for: Association of Staphylococcal Populations on Teatcups of Milking Parlours with Vaccination against Staphylococcal Mastitis in Sheep and Goat Farms
Source: Pathogens. 2021 Mar 24;10(4):385. doi: 10.3390/pathogens10040385 (PMC8063832; doi:10.3390/pathogens10040385)
Supplement: Supplementary file 1 [file pathogens-10-00385-s001.pdf]

Staphylococcal Populations on Teatcups of Milking Parlours in Sheep and Goat Farms and Associations with Vaccination Against Staphylococcal Mastitis

Charalambia K. Michael, Daphne T. Lianou, Natalia G.C. Vasileiou, Katerina Tsilipounidaki, Angeliki I. Katsafadou, Antonis P. Politis, Nikos G. Kordalis, Katerina S. Ioannidi, Dimitris A. Gougoulis, Constantina Trikalinou, Denise C. Orfanou, Ilektra A. Fragkou, Panagiota I. Kontou, Dimitra V. Liagka, Vasia S. Mavrogianni, Efthimia Petinaki and George C. Fthenakis

Table S1. Identity of staphylococcal isolates recovered from teatcups of milking parlours in sheep flocks (*n*=12) during a longitudinal study (4 visits) and their frequency of recovery.

**Upper part of teatcups (*n*=39)**		**Lower part of teatcups (*n*=17)**	
Species	n	Species	n
	1st visit		
S. aureus	2	*S. equorum*	1
S. capitis	1	*S. haemolyticus*	1
S. equorum	1	*S. lentus*	1
S. haemolyticus	1		
S. lentus	1		
S. sciuri	1		
S. simulans	1		
	2nd visit		
S. aureus	1	*S. aureus*	1
S. capitis	2	*S. capitis*	1
S. equorum	2	*S. equorum*	1
S. lentus	1	*S. lentus*	1
S. pettenkoferi	1		
S. saprophyticus	1		
S. sciuri	1		
S. simulans	1		
	3rd visit		
S. aureus	1	*S. aureus*	1
S. capitis	1	*S. capitis*	1
S. equorum	4	*S. xylosus*	1
S. lentus	2		
S. sciuri	1		
S. warneri	1		
S. xylosus	1		
	4th visit		
S. aureus	1	*S. aureus*	1
S. capitis	1	*S. equorum*	2
S. equorum	2	*S. lentus*	2
S. lentus	2	*S. sciuri*	1
S. saprophyticus	1	*S. simulans*	1

S. sciuri	1
S. simulans	2

Table S2. Summary of characteristics of 321 farms included into a cross-sectional study in Greece regarding staphylococcal populations on teatcups of milking parlours.

Geographical part of the country, where farms were located [1]	n
Central part of the country	134
Islands of the country	36
North part of the country	90
South part of the country	61
**Management system applied in the farms [2]**	**n**
Intensive management system	52
Semi-intensive management system	158
Semi-extensive management system	106
Extensive management system	5
**Animal species farmed**	**n**
Sheep	255
Goats	66
**Number of female animals in the farms**	**n**
<165	91
166-330	106
331-500	71
501-830	38
>830	15

[1] Central part: includes the administrative regions of Continental Greece and Thessaly, as well as the division of Aetolia-Acarnania of the administrative region of Western Greece and the divisions of Arta and Preveza of the administrative region of Epirus; Islands part: includes the administrative regions of Crete, Ionian islands, North Aegean and South Aegean; North part includes the administrative regions of Central Macedonia, Eastern Macedonia and Thrace and Western Macedonia, as well as the divisions of Ioannina and Thesprotia of the administrative region of Epirus; South part: includes the administrative regions of Attica and Peloponnese, as well as the divisions of Achaea and Elis of the administrative region of Western Greece.

[2] Classification according to the European Food Safety Authority system (European Food Safety Authority. Scientific opinion on the welfare risks related to the farming of sheep for wool, meat and milk production. *EFSA J.* **2014**, *12*, 3933-4060).

Table S3. Summary of characteristics of 12 sheep farms included into a longitudinal study in Greece regarding staphylococcal populations on teatcups of milking parlours.

Geographical part of the country, where farms were located [1]	n
Central part of the country	0
Islands of the country	0
North part of the country	0
South part of the country	12
**Management system applied in the farms [2]**	**n**
Intensive management system	1
Semi-intensive management system	8
Semi-extensive management system	3
Extensive management system	0
**Number of female animals in the farms**	**n**
<165	3
166-330	8
331-500	0
501-830	1
>830	0

[1] Central part: includes the administrative regions of Continental Greece and Thessaly, as well as the division of Aetolia-Acarnania of the administrative region of Western Greece and the divisions of Arta and Preveza of the administrative region of Epirus; Islands part: includes the administrative regions of Crete, Ionian islands, North Aegean and South Aegean; North part includes the administrative regions of Central Macedonia, Eastern Macedonia and Thrace and Western Macedonia, as well as the divisions of Ioannina and Thesprotia of the administrative region of Epirus; South part: includes the administrative regions of Attica and Peloponnese, as well as the divisions of Achaea and Elis of the administrative region of Western Greece.

[2] Classification according to the European Food Safety Authority system (European Food Safety Authority. Scientific opinion on the welfare risks related to the farming of sheep for wool, meat and milk production. *EFSA J.* **2014**, *12*, 3933-4060).

Table S4. Details of vaccines recorded to be in use in Greece against staphylococcal mastitis, during a countrywide investigation in sheep and goat farms.

Vaccine	Commercial name	Animal species for which licenced	Immunological component active against staphylococcal mastitis [1]
I	Mastivet	Cattle	$\geq 10^8$ c.f.u. inactivated whole-cell *S. aureus*
II	Ovax	Sheep	160 c.f.u. inactivated whole-cell *S. aureus*<br>a-staphylococcal toxoid 120 HU<br>b-staphylococcal toxoid 2000 HU
III	Vimco, Startvac	Cattle, Sheep, Goats	Bacterin of *S. aureus* strain, expressing the exopolysaccharide poly-N-acetylglucosamine (PNAG)
IV	-	-	*S. aureus* autogenous vaccine prepared with an isolate from clinical mastitis diagnosed in animals of the farm

[1] Information drawn from the respective Summary of Product Characteristics
